# The effect of propolis supplementation on clinical symptoms in patients with coronavirus (COVID-19): A structured summary of a study protocol for a randomised controlled trial

**DOI:** 10.1186/s13063-020-04934-7

**Published:** 2020-12-03

**Authors:** Mahsa Miryan, Davood Soleimani, Leila Dehghani, Karim Sohrabi, Farzin Khorvash, Mohammad Bagherniya, Sayed Mazaher Sayedi, Gholamreza Askari

**Affiliations:** 1 Student Research Committee, Tabriz University of Medical Sciences, Tabriz, Iran; 2 Nutrition Research Center, Department of Clinical Nutrition, School of Nutrition and Food Sciences, Tabriz University of Medical Sciences, Tabriz, Iran; 3 Nutritional Sciences Department, School of Nutrition Sciences and Food Technology, Kermanshah University of Medical Sciences, Kermanshah, Iran; 4 Department of Tissue Engineering and Regenerative Medicine, School of Advanced Technologies in Medicine, Shahid Beheshti University of Medical Sciences, Tehran, Iran; 5 Department of Emergency Medicine, Alzahra University Hospital, Isfahan University of Medical Sciences, Isfahan, Iran; 6 Department of Infectious Diseases, Faculty ofMedicine, Nosocomial Infections Research Center, Isfahan University of Medical Sciences, Isfahan, Iran; 7 Food Security Research Center, Isfahan University of Medical Sciences, Isfahan, Iran; 8 Anesthesia and Critical Care Research Center, Isfahan University of Medical Sciences, Isfahan, Iran; 9 Department of Community Nutrition, School of Nutrition and Food Science, Isfahan University of Medical Sciences, Isfahan, Iran; 10 Honey Bee Expert of Isfahan Agricultural and Natural Resources Research Center, Arib Ave., Isfahan, 81746-73461 Iran

**Keywords:** COVID-19, Coronavirus, Protocol study, Propolis, Randomised controlled trial

## Abstract

**Objectives:**

This study aims to assess the effect of propolis supplementation on clinical symptoms in patients with coronavirus (COVID-19).

**Trial design:**

This is a Double-Blind, Placebo-Controlled, Parallel Arm, Randomized Phase ΙΙ Clinical Trial.

**Participants:**

Patients with the confirmed COVID-19 based on the PCR test are eligible to participate in the trial if they are 18 to 75 years of age and have no history of the current use of warfarin or propolis supplement and presence of sensitivity to bee products. Patients will be recruited from the Al-Zahra hospital in Isfahan city, Isfahan, Iran.

**Intervention and comparator:**

Participants (N=40) in the intervention group will receive an identical propolis tablet (containing 300 mg Iranian green propolis extract) three times a day for a period of 2 weeks. Participants (N=40) in the control group will receive an identical placebo tablet (containing 300 mg microcrystalline cellulose) three times a day for 2 weeks. All tablets are prepared by the Reyhan Naghsh Jahan Pharmaceutical Co., Isfahan, Iran.

**Main outcomes:**

The main outcomes are changes in the coronavirus disease’s clinical symptoms including duration and severity from baseline to the end of 2 weeks.

**Randomization:**

Eligible patients will be randomly allocated in a 1:1 ratio to the intervention or control group. Randomization will be performed on the basis of permuted block sizes of 4 and will be stratified according to sex categories. Randomization sequences will be prepared by the trial’s pharmacist with the use of random-number tables.

**Blinding (masking):**

The trial-group assignment will be concealed from all participants, clinicians, and investigators throughout the trial. To ensure blinding, randomization sequences will be kept in identical, opaque, sealed, sequentially numbered envelopes. Only the trial's pharmacist has access to the randomization list. Also, the placebo tablet will be similar to the propolis tablet in terms of texture, taste, color, odor, and weight. Both tablets will be provided in containers that are completely identical in weight, shape, labelling, and packaging.

**Numbers to be randomized (sample size):**

The calculated total sample size is 80 patients, with 40 patients in each group.

**Trial status:**

The protocol is Version 1.0, October 10, 2020. Recruitment began August 22, 2020, and is anticipated to be completed by March 21, 2021.

**Trial registration:**

The name of the trial register:

The effect of propolis supplementation on clinical symptoms in patients with coronavirus (COVID-19): A randomized, double-blind, placebo-controlled clinical trial.

IRCT registration number:

IRCT20200802048267N1.

Date of trial registration:

20 October 2020, retrospectively registered.

**Full protocol:**

The full protocol is attached as an additional file, accessible from the Trials website (Additional file 1). In the interest of expediting the dissemination of this material, the familiar formatting has been eliminated; this Letter serves as a summary of the key elements of the full protocol.

**Supplementary Information:**

The online version contains supplementary material available at 10.1186/s13063-020-04934-7.

## Supplementary Information

**Additional file 1.** Full Study Protocol.

## Data Availability

The final dataset of the trial will be available upon request from the primary investigator via e-mail at askari@mui.ac.ir, after obtaining the permission of the Regional Ethics Committee.

