# Supplementary material for: The effect of propolis supplementation on clinical symptoms in patients with coronavirus (COVID-19): A structured summary of a study protocol for a randomised controlled trial
Source: Trials. 2020 Dec 3;21:996. doi: 10.1186/s13063-020-04934-7 (PMC7713667; doi:10.1186/s13063-020-04934-7)
Supplement: Supplementary file 1 — Additional file 1. Full Study Protocol. [file 13063_2020_4934_MOESM1_ESM.docx]

**بررسی اثر مکمل بره موم زنبور عسل بر علائم بالینی در بیماران مبتلا به ویروس کووید-19: یک مطالعه کارآزمایی بالینی تصادفی شده دوسوی کور و کنترل شده با دارونما**

**مقدمه**

**بیان مسئله**

کروناویروس‌ها (نام علمی: Coronaviruses) خانواده بزرگی از ویروس‌ها و زیر مجموعه کرونا ویریده هستند که از ویروس سرماخوردگی معمولی تا عامل بیماری‌های شدیدتری همچون سارس، مرس و کووید ۱۹ را شامل می‌شود. آنها موجب بروز بیماری هایی از خانواده سرما خوردگی در انسان و حیوانات می‌گردند و از ویژگی ظاهری آن‌ها زوائد تاج مانند بر روی سطوح دیواره ویروس‌ها می‌باشد. گاهی برخی از کروناویروس‌ها به دستگاه تنفسی حمله می‌کنند و گاهی علائم خود را در روده و معده افراد نمایان می‌کنند. علائم ویروس کرونا در ریه، معمولاً در سرماخوردگی‌های معمول و نوعی سرماخوردگی ترشح آور به نام پِنومونیا ظاهر می‌شود که معمولاً در بیشتر افراد با سرماخوردگی ملایمی همراه است (1-3).

در دسامبر ۲۰۱۹ ، برای اولین بار در شهر ووهان استان هوبئی^[[1]](#footnote-1)^ چین، پس از اینکه مردم بدون علت مشخصی دچار سینه پهلو شدند و واکسن‌ها و درمان‌های موجود مؤثر نبودند، نوع جدیدی از کروناویروس با همه‌گیری در انسان شناسایی شد (3). تا ۳۰ بهمن، این کروناویروس جدید، جان بیش از ۲۷۰۹ نفر را گرفته و بیش از ۷۳٬۳۳۲ نفر به این ویروس در ۸۰ کشور تأیید شده که از جمله در تایلند، کره جنوبی، ژاپن، تایوان، استرالیا، سنگاپور، نپال، ویتنام، اندونزی، آلمان، روسیه، فیجی، فرانسه، ایران و آمریکا مبتلا شدند (4). با عبور تعداد قربانیان ویروس کرونا از مرز ۱۰۰۰ نفر سازمان جهانی بهداشت برای بیماری ناشی آن نام رسمی انتخاب کرده‌است. کووید-۱۹ که اشاره‌ای دارد به «کرونا»، «ویروس»، «بیماری» و سال ۲۰۱۹ این نوع کرونا ویروس قبل از این همه‌گیری در انسان دیده نشده بود (5). برآوردهای نخستین نشان می‌دهد که درصد مرگ و میر این ویروس بین ۲ تا ۳ درصد مبتلا شدگان است (4). علایم بیماری شامل تب، سرفه های خشک و گاهی مشکلات تنفسی مانند تنگی نفس، تندنفسی و گلودرد و آبریزش بینی است (6-8). روش های تشخیص این بیماری علاوه بر بررسی علائم حیاتی بیمار، گرافی و اسکن از ریه ها (HRCT)، نمونه گیری از مخاط حلق و بینی جهت بررسی با PCR و اورال-فکال می باشد (9).

مشکلات تنفسي بطور سنتي و از گذشته، توسط بره موم بطور موثري درمان مي‌شدند و محققان داروسازی اخيراً نقش بره موم در مهار روند التهاب در مشکلات تنفسي را به تصوير کشيده‌اند (10). در سال 1975 میهالیچکو^^[[2]](#footnote-2)^^ محقق اهل روماني 30 بيمار که از آسم برونشیتی رنج مي‌بردند را درمان نمود. بيمارن را به دو گروه تقسيم کردند، يک گروه تحت درمان‌هاي معمول و گروه ديگر تحت درمان با بره موم قرار گرفتند. گروهي که با بره موم درمان شدند بهبود بيشتري را نسبت به گروه ديگر نشان دادند (11-13). دکتر مک ایوان متخصص آلرژي، استفاده از دوزهاي بالاي بره موم براي بيماران آسمي که از عفونت هاي متداول قفسه سينه‌ی رنج مي‌بردند و بطور معمول نيازمند درمان با آنتي‌بيوتيک‌ها بودند را پيشنهاد داد. بره موم به محض احتمال بروز سرماخوردگي يا عفونت بايد مصرف شود. او پيشنهاد داد که درمان به مدت پنج روز ادامه يابد. در مورد بيماري‌هاي مزمن که فرد بايد بطور اضطراری در بيمارستان بستري شود، وي درمان طولاني مدت با بره موم را پيشنهاد ‌داد. تحقيقات باليني در بخش آسم بيمارستان عمومي بارنزلی در بريتانيا در حال انجام است(14). اثر پیشگیری کنندگی بره موم در سال 1972 در تحقیق شوچنکو^^[[3]](#footnote-3)^^ روسی به اثبات رسید. در این تحقیق مشخص شد که تغذیه‌ی موش با بره موم قبل از تماس با آنفولانزا، وی را در مقابل آن ایمن می‌ساخت، ولی اگر مبتلاء می‌شدند، تاثیری نداشت. مشابه این تحقیق در رومانی و بلغارستان، اثر پیشگیری کنندگی بره موم در جلوگیری از ابتلاء موش‌ها به ویروس آنفولانزا را اثبات کرد. مهمترین آزمایش در مورد تاثیر بره موم بر علیه آنفولانزا، در سال 1978 در اپیدمی این بیماری، در شهر سارایوو یوگسلاوی صورت گرفته است. در طی این اپیدمی پروفسور اسماناژیک^^[[4]](#footnote-4)^^ تحقیقی روی دانشجویان یک دانشکده پرستاری که در معرض بیماری آنفولانزا بودند انجام داد.80 دانشجو بره موم را دریافت کردند و 182 نفر آن را مصرف نکردند. از آنهایی که بره موم دریافت کردند فقط 7% بیمار شدند در حالی که 63% از آنهایی که مصرف نکرده بودند بیمار شدند. در تحقیق دیگری در سال 1978 در یوگسلاوی^[[5]](#footnote-5)^، نتایج مشابهی گزارش شد (15).

بین سال‌های 1994 تا 1997، سازمان مشاوره بهداشت در نایروبی^[[6]](#footnote-6)^ (بخشی از طرح کنترل بین‌المللی ایدز)، کپسول‌های بره موم را برای افرادی که مبتلاء به ایدز یا ویروس HIV و عفونت كامل AIDS بودند، فراهم کردند. در مقاله‌ای کوتاه نويسنده بهبود، سلامت عمومي و افزایش اشتها ميان آسيب ديدگان كه در بعضي از موارد منجر به از سرگيري زندگي طبيعي و بازگشت به كار شده بود گزارش نمود. در سال 1975، دورشنكو^^[[7]](#footnote-7)^^، در بیمارستان شهر کیف (اتحاد جماهیر شوروی سابق)، 238 بیمار مبتلاء به التهاب حلق را با مخلوط بره موم درمان کرد. او یک سهم از عصاره‌ی 30% بره موم را با گلیسیرین (یا روغن هلو) مخلوط می‌کرد و بر غشای مخاطی در ناحیه عفونی به مدت بیش از 10 تا 15 روز قرار می‌داد. 75% بیماران بطور کامل بهبود یافتند، در حالی‌که 14% اثری ندیدند. محققین دریافتند که عصاره‌ی بره موم اثر داروهای رایج در بازار براي درمان التهاب حلق را افزايش مي‌دهد (16). محققین رومانیایی همان سال بره موم را برای بیش از 200 بیمار مبتلاء به التهاب حاد حلق، التهاب حاد حنجره و التهاب حاد لوزه مورد استفاده قرار دادند. در هر گروه درمانی10% از بیماران با روش رایج درمان شدند. بیماران درمان شده با بره موم بسیار سریع‌تر از گروه کنترل بهبود یافتند (14, 16). ترکیب سازنده بره موم عمدتا شامل استر اسيدهاي چرب و الكل‌هايي با زنجير طولاني هيدروكربني مي‌باشد كه غالبا از لحاظ شيميايي غير فعال هستند و در عين حال به طور گسترده در مواد آرايشي به كار مي‌روند. هرچند، در مصارف پزشکی بره موم از عصاره‌ی آن استفاده می‌شود که موم آن جدا شده است. رايحه‌ي بره موم عمدتاً ناشي از مواد فرار موجود در آن بويژه ترپن‌ها مي‌باشد. در مقام مقايسه، بره موم حاصله از مناطق گرمسيري داراي مقادير كمتري از اين مواد مي‌باشد و اطلاعات ما درباره‌ي فعاليت بيولوژيكي آنها بسيار كم است. بره موم در مناطق استوايي داراي مقادير زيادتري از اين مواد مي‌باشد و فعاليت‌هاي بيولوژيكي در آنها شناخته شده‌تر است. ترکیبات پلی فنلی بره موم نیز عمدتا شامل استر فنیل اتیل كافئيك يا CAPE مي‌باشند كه بنابر تحقيقات انجام يافته در بروز تعدادي از خواص دارويي نيز نقش دارند. پينوسمبرين و گالانژين از دیگر ترکیب پلی فنولی در بره موم می باشد که و بيشترين اهميت را در تركيب بره موم دارا هستند. توجه به اين نكته كه فلاونوئيدها در گياهان تغييرات بيوشيميايي ظريفي را در هنگام ظهور در بره موم از خود نشان مي‌دهند مهم است و اين امر متعاقب فعل و انفعالات آنزيمي غدد بزاقي زنبور صورت مي‌گيرد. مقادير فلاونوئيدها در بره موم طيفي از10 تا 20 درصد را شامل مي‌شود و بزرگترين گروه مستقل بيوشيميايي را در بره موم تشكيل مي‌دهند. همچنين محتويات فلاونوئيدي بره موم است كه نقطه‌ي اشتراك علاقه‌ي محققان محسوب می‌شود.

فلاونوئيدها در بره موم اروپايي، تشكيل دهنده‌ي بيش از 20 درصد از وزن آن مي‌باشند. تفاوت اساسي بين فلاونوئيدهاي موجود در گياهان و در بره موم این است که‌، آن دسته كه در بره موم موجود مي‌باشند گليكوزيدي نمي‌باشند بدين معني كه بر خلاف آنچه كه در گياهان وجود دارد اين دسته داراي تركيبات قندي متصل به ساختار بيوشيميايي نيستند. اين تفاوت به واسطه فرایند صورت گرفته از طرف زنبور عسل بر روي فلاونوئيدها و از طريق ترشح آنزيم‌ها می‌باشد. حقيقت آن است كه آن‌ها تغيير می‌یابند و اين خود مي‌تواند توضيحي مختصر براي خواص درماني منحصر به فرد و پايدار بره موم باشد (17). بررسی‌ در ارتباط با وجود فلاونوئيدها در ديگر محصولات كندوي زنبور عسل از جمله عسل و گرده صورت گرفته است. ساباتير و دیگران در دانشگاه کویمبرا^[[8]](#footnote-8)^ كشور پرتغال در سال 1995 دریافتند كه محتويات فلاونوئيد موجود در گرده 05/0% ، در عسل 006/0 % و در بره موم 10 % مي‌باشد.

گزارش‌هاي كلينيكي مبين آن است كه درمان با بيوفلاونوئيدها در بیماریهای مختلف از جمله تب روماتيسمي، سقط‌ جنین‌های خود بخود و غير خود بخود، فشار خون بالا، عفونت‌هاي ریوی، هموروئيدها و سيروز كبدي موثر است. در دهه‌ي 1990 علاقه‌ي علمي به اين موضوع بطور روز افزون افزايش يافت. بيوفلاونوئيدهاي موجود در بره موم داراي اثر حفاظتي بر عليه عفونت حاصل از ويروس‌ها هستند. در سطح خارجي ويروس‌ها، يك غشاي پروتئيني قرار گرفته است. مادامي كه اين غشاء، تجزيه نشده باقي بماند، عفونت و مواد خطرناك آن‌ها محفوظ مانده و در اين حالت براي انواع ارگانيسم‌ها مضر نیستند. ما آنزيمي كه غشاي پروتئيني ويروس‌ها را جدا مي‌كند، در حالي يافتيم كه مهار شده بود. بنابراين ماده ويروسي خطرناك در آن محفوظ می‌ماند. غشاي پروتئيني اطراف ويروس به واسطه‌ی بيوفلاونوئيدها در بره موم حفظ مي‌شود، فلاونوئيدها، ويروس را كاملا غير فعال نگاه مي‌دارند. اين مسئله درست مانند آن است كه ايمني بر عليه ويروس صورت گرفته باشد اما اين امر تنها به واسطه حضور بيوفلاونوئيدها در بره موم رخ می‌دهد. اين مسئله براي ما تصويري جذاب از نقش فلاونوئيدها در بره موم ايجاد مي‌كند.

تحقيقات دكتر هاوستين^[[9]](#footnote-9)^ در آلمان را بياد مي‌آوريم كه نشان داد چگونه فلاونوئيدها از تجزيه‌ی غشاي پروتئين ويروس‌ها به واسطه‌ی مهار فعاليت آنزيمي و ممانعت از ورود آنها و در نتيجه‌ی جلوگيري از بروز عفونت در ارگانيسم عمل مي‌نمايند**.**

از هم گسيختگي غشا سلول در فرايند التهاب منجر به رهاسازي اسيدهاي چرب منجر به توليد اسيد آراشيدونيك منجر به توليد پروستاگلاندين‌ها و لكوترين‌ها كه باعث تراوش از مويرگ‌هاي خوني قرمزي پوست**،** آزاد شدن هيستامين درد و تجمع مايعات مي شود.

فرآيند التهاب يك عامل كليدي، در بيماري‌هاي ناتوان كننده شامل آسم، پسوريازيس، سندرم دیسترس تنفسي بالغين، التهاب آلرژيك بيني، نقرس، روماتوئيد آرتريت، ميگرن، بيماري هاي التهابي روده، التهاب لثه و زخم هاي دهان است.

تحقيقات بکار گرفته شده توسط آموروس^^[[10]](#footnote-10)^^ در دانشکده‌ي داروسازي رن فرانسه در سال 1992 تناقض يافته‌هاي بالا را بيان کرد. بره موم همانند تعدادي از فلاونوئيدهاي خاص و فلاون‌ها به وضوح نشان داد که از رشد تعدادي از ويروس شامل ويروس عامل پلی میلیت و هرپس ویروس‌های ساده جلوگيري مي‌کند. مشخص شده است كه بعضی از فلاون‌ها اثر بيشتري نسبت به بقيه دارند كه ترتيب آن عبارت است از: گالانزین، کامپفرول، کوئرستين. علاوه بر اثر بر روي تکثير ويروس‌ها همچنين متوجه شدند که بره موم يک تاثير ويروس‌کشي بر روي ويروسهاي HSV و VSV دارد. اثرات تركيبات بره موم شرح مي‌دهد كه چرا بره موم فعال‌تر از اجزاء مجزاي آن است. اثرات سینرزیتی (همپوشانی) کلیه ترکیبات بره موم دلیل فعال‌تر بودن آن نسبت به اجزاء منفرد آن را توجیه می‌کند. درسال 1992 يک محقق بلغاري بنام سرکدجیوا^^[[11]](#footnote-11)^^ بره موم و برخي از اجزايش را روي محيط کشت‌هاي آلوده با ويروس‌هاي آنفولانزای هنگ کنگي امتحان کرد. بيشترين تاثير وقتي بدست آمد كه بره موم قبل و بعد از آلودگي به کاربرد شده بود. در اين آزمایش، مشتق فنوليک اسيد در بره موم نشان داد که فعال است (18-20).

با توجه به شیوع ویروس کرونا و همه گیر شدن بیماری کوید 19 و همچنین بروز خسارات جانی ناشی از آن می طلبد تا راه های مقابله با این بیماری را جستجو کنیم. استفاده از فرآورده های طبیعی از جمله بره موم زنبور عسل که سابقه آن در درمان بیماری های عفونی و ویروسی بسیار زیاد است ما را بر آن داشت تا این طرح را تنظیم و اجرا نماییم**.**

**روش اجرا**

*نوع مطالعه و روش پژوهش*

کارآزمایی بالینی، تصادفی شده، دوسویه کور و کنترل شده با دارونما

*جامعه آماری پژوهش*

این مطالعه بر روی 80 بیمار بالغ 20 تا 75 ساله مبتلا به ویروس کرونا در بیمارستان های وابسته به دانشگاه علوم پزشکی اصفهان در سال 1399.

*معیارهای ورود به مطالعه*

- تمایل به شرکت در مطالعه و پرکردن فرم رضایت آگاهانه
- سن20-75 سال
- تشخیص ابتلا به بیماری COVID-19براساس یافته های حاصل از تصویربرداری (Chest X Ray)، سی تی اسکن و یا PCR^^[[12]](#footnote-12)^^

معیارهای *عدم ورود به مطالعه*

- *مصرف داروی وارفارین*
- *حساسیت به محصولات زنبور عسل و یا فرآورده های گیاهی*

معیارهای خروج از مطالعه

- *عدم تمایل به ادامه همکاری (افرادی که از مطالعه خارج میشوند درمان اصلی خود را دریافت کرده و تحت نظر می باشند. این درمان بعنوان یک درمان مکمل می باشد که در طرح ذکر شده است)*
- *مشاهده هرگونه عوارض جانبی، ایجاد هر یک از شرایط عدم ورود در حین مطالعه*
- *عوارض جانبی که می توانند پس از مصرف مکمل موجب توقف طرح یا خروج آزمودنی از مطالعه شوندکه شامل مشکل گوارشی ناخواسته (دل درد، دل پیچه، تهوع، استفراغ، اسهال یا غیره)، حساسیت آلرژیکی (اعم از پوستی، تنفسی یا غیره) و یا ایجاد هر گونه عارضه نامطلوبی که تا قبل از مصرف مکمل وجود نداشته است. شایان ذکر است که تا کنون در مطالعات بالینی در این دوز اثر سوی از این مکمل گزارش نشده است.*
- *کاهش سطح هوشیاری و نیازمندی به تغذیه از راه لوله^^[[13]](#footnote-13)^^*

این مطالعه، یک کارآزمایی بالینی تصادفی دوسوکور است که بر روی 80 بیمار مبتلا به ویروس کووید-19 انجام خواهد شد. شرکت کنندگان این مطالعه مبتلایان به ویروس کووید-19 که در روز اول بستری در بیمارستان می باشد، دعوت بعمل می آید. در این مطالعه به منظور کاهش ناهمگونی بین گروه های مورد مطالعه از طرح بلوک های طبقه بندی شده^^[[14]](#footnote-14)^^ جهت تصادفی سازی تخصیص شرکت کنندگان به گروه های مورد مطالعه استفاده می گردید. در این مطالعه تمامی شرکت کنندگان پس از کسب معیارهای شایستگی و رضایت نامه کتبی بر اساس طبقه جنسیت و شدت بیماری با بلوک سازهای چهارتایی به نسبت مساوی به دو گروه کنترل و مداخله تقسیم خواهند شد. در این مطالعه گروه مداخله در کنار رژیم غذایی روزانه سه عدد قرص 300 میلی گرمی بره موم (قبل از وعده صبحانه، ناهار و شام) را به مدت 14 روز دریافت می نمایند. در مقابل گروه کنترل نیز به طور مشابه در کنار رژیم غذایی، روزانه 3 عدد قرص پلاسبو که از نظر رنگ، بو و شکل شبیه دارو می باشند را دریافت خواهند نمود و به غیراز داروساز که وظیفه تهیه مکمل ها و توالی تصادفی سازی را بر عهده دارد، هیچکدام از شرکت کنندگان و دیگر محققین تا پایان مطالعه و انجام آنالیزهای آماری از آن آگاهی نخواهند داشت. این مطالعه به منظور پنهان سازی تخصیص تصادفی از قوطی های کدگذاری شده^^[[15]](#footnote-15)^^ استفاده می گردد. از شرکت کنندگان و محقیقین تا پایان مطالعه پنهان خواهد ماند. میزان تبعیت افراد بستری از مداخله تخصیص یافته شده از طریق بررسی کاردکس دارویی بخش بستری شده انجام خواهد شد. در مورد بیماران سرپایی نیز با تحویل جعبه خالی داروها، استفاده از چک لیست و تماس تلفنی میزان تبعیت افراد بررسی خواهد شد.

در ابتدا و انتهای مطالعه سطح شاخص های آنتی اکسیدانی- اکسیدانی بدن (SOD^^[[16]](#footnote-16)^^, MDA^^[[17]](#footnote-17)^^, TAC^^[[18]](#footnote-18)^^) فاکتورهای التهابی بدن (ESR & hs-CRP)، آنزیم های کبدی و شاخص های کلیوی، میزان گلبول های سفید خون، آلبومین فشار خون و شاخصه های بالینی مانند شدت سرفه، بهبودی گرافی، درجه حرارت بدن (تب)، میزان تنفس در دقیقه، آبریزش بینی، میزان و شدت سرفه و طول مدت بستری بیماری مورد ارزیابی قرار خواهد گرفت.

جمع آوری داده ها

تمامی شاخص ها فوق الذکر طبق پرسش نامه ضمیمه توسط یک نفر پزشک (برای هر بیمار یک پزشک مشخص برای قبل و بعد بیماری) در نظر گرفته می شود. در ضمن تعداد پزشکانی که این موارد را ارزیابی می کنند محدود می باشد و پزشک متخصص عفونی تیم تحقیق قبل از شروع طرح تمامی هماهنگی های لازم با پزشکانی که قرار است ارزیابی ها را انجام دهند صورت می پذیرید تا همگی معیار مشخصی را برای پر کردن پرسش نامه ها در نظر بگیرند و بصورت یکسان عمل کنند.

**1- ویژگی های دموگرافیک**

متغیرهای دموگرافیک (شامل سن، جنس، وضعیت تاهل، استعمال دخانیات، سابقه ی بیماری و تاریخچه پزشکی، سطح تحصیلات، شغل، مصرف مکمل ها و داروها) با تکمیل پرسشنامه اطلاعات عمومی از تمامی شرکت کنندگان در ابتدای مطالعه جمع آوری خواهد شد.

**2- اندازه گیری فشار خون**

فشارخون به وسیله فشارسنج جیوه ای کالبیر شده پس از 10 دقیقه نشستن بیمار در دو مرحله به فاصله حداقل 5 دقیقه اندازه گیری خواهد شد و میانگین دوبار اندازه گیری به عنوان فشار خون نهایی لحاظ می گردد. قبل از گرفتن فشار خون، از بیماران در مورد استعمال سیگار یا مصرف قهوه در 2 ساعت قبل سوال خواهد شد.

3- شدت سرفه

برای تعیین شدت سرفه از مقیاس آنالوگ بصری^[[19]](#footnote-19)^ (VAS)استفاده خواهد شد.

**4- اندازه گیری های بیوشیمیایی**

در این روش از بیماران در حالت ناشتایی 5 سی سی نمونه خون گرفته خواهد شد. میزان آنزیم های کبدی سرم با استفاده از روش فتومتریک آنزیماتیک با کیت کلریمتریک و با استفاده از دستگاه اتو آنالایزر اندازه گیری خواهد شد و همچنین سطوحhs-CRP^^[[20]](#footnote-20)^^ نیز با استفاده از کیت الایزا اندازه گیری خواهد شد. شاخص های استرس اکسیداتیو ظرفیت تام آنتی اکسیدانی-اکسیدانی به روش کالریمتریک و با استفاده از کیت های تجاری کیازیست (تهران، ایران) اندازه گیری خواهد شد.

**5- تصویربرداری از ریه ها**

قبل و بعد از مداخله از بیماران تصویربرداری (Chest X Ray) و سی تی اسکن گرفته می شود و وضعیت ریه آن ها قبل و بعد از مداخله توسط متخصص عفونی طرح بررسی می شود. بهبود وضعیت تصویر ریه ها از مهمترین علائم بالینی طرح می باشد.

آنالیزهای آماری

داده ها در ابتدا توسط آزمون کولموگروف-اسمیرنوف و نمودار Q-Q plot مورد ارزیابی نرمالیته انجام خواهد. دادهای با توزیع نرمال به صورت به صورت میانگین (انحراف معیار)، غیر نرمال میانه (دامنه میان چارکی) و متغیرهای کیفی به صورت تعداد (درصد) گزارش خواهند شد. با توجه به نقش التهاب در این بیماران، به منظور تشخیص تفاوت اثر استاندارد شده به اندازه 7/0 در شاخص hs-CRP و با در نظر گرفتن سطح معنی داری 5 درصد (آلفا) و توان 80 درصد (بتا)، تعداد 34 نفر در هر گروه تعیین گردید و باتوجه به ماهیت پیگرانه و ریزش 15 درصد در هر گروه 40 نفر تعیین گردید (21). تحلیل های درون گروهی با استفاده از آزمون تی زوجی یا ویلکاکسون و تحلیل های بین گروهی با استفاده از آزمون تی مستقل و یا من-ویتنی انجام خواهد شد. برای توزیع متغیرهای کیفی با استفاده از آزمون *کای دو* بین دو گروه مقایسه خواهد شد. همچنین برای تعدیل اثر مخدوشگرها از جمله شدت بیماری، سن و جنس بیماران از آنالیز کوواریانس^^[[21]](#footnote-21)^^ استفاده خواهد شد. در این مطالعه به منظور برآورد تعدیل اثر ریزش نمونه ها در طی مطالعه، از روش تحلیل با قصد درمان (ITT) ^^[[22]](#footnote-22)^^با جایگزینی داده های گمشده با آخرین مقدار مشاهده شده^^[[23]](#footnote-23)^^ (LOCF)استفاده می گردد. جهت تحلیل داده ها از نرم افزار SPSS ورژن 16 به منظور تحلیل داده ها با سطح معناداری کمتر از 5 درصد استفاده خواهد شد.

**References:**

1. Jiang S, Shi Z, Shu Y, Song J, Gao GF, Tan W, et al. A distinct name is needed for the new coronavirus. Lancet (London, England). 2020;395(10228):949.

2. Corman VM, Muth D, Niemeyer D, Drosten C. Hosts and sources of endemic human coronaviruses. Advances in virus research. 100: Elsevier; 2018. p. 163-88.

3. Rajabian M, Doagooyan M. Coronavirus Disease 2019 (Covid-19) in Middle East: Data, Facts and Doubts. International Journal of Pediatrics. 2020:11429-34.

4. Mansoor S, Kelly S, Murphy K, Waters A, Siddiqui NS. COVID-19 pandemic and the risk of infection in multiple sclerosis patients on disease modifying therapies:“what the bleep do we know?”. The Egyptian Journal of Neurology, Psychiatry and Neurosurgery. 2020;56:1-3.

5. Sarkar B, Ullah MA, Johora FT, Taniya MA, Araf Y. The Essential Facts of Wuhan Novel Coronavirus Outbreak in China and Epitope-based Vaccine Designing against 2019-nCoV. BioRxiv. 2020.

6. Organization WH. Laboratory biosafety guidance related to coronavirus disease 2019 (COVID-19): interim guidance, 12 February 2020. World Health Organization; 2020.

7. Peeri NC, Shrestha N, Rahman MS, Zaki R, Tan Z, Bibi S, et al. The SARS, MERS and novel coronavirus (COVID-19) epidemics, the newest and biggest global health threats: what lessons have we learned? International journal of epidemiology. 2020.

8. Lai C-C, Shih T-P, Ko W-C, Tang H-J, Hsueh P-R. Severe acute respiratory syndrome coronavirus 2 (SARS-CoV-2) and corona virus disease-2019 (COVID-19): the epidemic and the challenges. International journal of antimicrobial agents. 2020:105924.

9. Organization WH. Laboratory testing for coronavirus disease 2019 (COVID-19) in suspected human cases: interim guidance, 2 March 2020. World Health Organization; 2020.

10. De Castro S. Propolis: biological and pharmacological activities. Therapeutic uses of this bee-product. Annual Review of Biomedical Sciences. 2001;3:49-83.

11. Banskota AH, Tezuka Y, Prasain JK, Matsushige K, Saiki I, Kadota S. Chemical constituents of Brazilian propolis and their cytotoxic activities. Journal of Natural Products. 1998;61(7):896-900.

12. Ghisalberti E. Propolis: a review. Bee world. 1979;60(2):59-84.

13. Mihaela B, Necula V, Puchianu G, Enache D. Researches on the Antimicrobial Impact of Imunosept Product. 2017.

14. Sforcin J. Propolis and the immune system: a review. Journal of ethnopharmacology. 2007;113(1):1-14.

15. Sy LB, Wu Y-L, Chiang B-L, Wang Y-H, Wu W-M. Propolis extracts exhibit an immunoregulatory activity in an OVA-sensitized airway inflammatory animal model. International immunopharmacology. 2006;6(7):1053-60.

16. Doroshenko P. Treatment of chronic tonsillitis patients with a propolis-wax paste. Meditsinskaia sestra. 1983;42(11):36.

17. Jung W-K, Lee D-Y, Choi YH, Yea SS, Choi I, Park S-G, et al. Caffeic acid phenethyl ester attenuates allergic airway inflammation and hyperresponsiveness in murine model of ovalbumin-induced asthma. Life Sciences. 2008;82(13-14):797-805.

18. Burdock G. Review of the biological properties and toxicity of bee propolis (propolis). Food and Chemical toxicology. 1998;36(4):347-63.

19. Amoros M, Simõs C, Girre L, Sauvager F, Cormier M. Synergistic effect of flavones and flavonols against herpes simplex virus type 1 in cell culture. Comparison with the antiviral activity of propolis. Journal of Natural Products. 1992;55(12):1732-40.

20. Serkedjieva J, Manolova N, Bankova V. Anti-influenza virus effect of some propolis constituents and their analogues (esters of substituted cinnamic acids). Journal of Natural Products. 1992;55(3):294-7.

21. Allen Jr JC. Sample size calculation for two independent groups: a useful rule of thumb. Proceedings of Singapore Healthcare. 2011;20(2):138-40.

1. Hubei [↑](#footnote-ref-1)
2. Mihailescu [↑](#footnote-ref-2)
3. Shevchenko [↑](#footnote-ref-3)
4. Osmanagic [↑](#footnote-ref-4)
5. Yugoslavia [↑](#footnote-ref-5)
6. Nairobi [↑](#footnote-ref-6)
7. Doroshenko [↑](#footnote-ref-7)
8. Coimbra [↑](#footnote-ref-8)
9. Havestin [↑](#footnote-ref-9)
10. Amoros [↑](#footnote-ref-10)
11. Serkedjeyona [↑](#footnote-ref-11)
12. Polymerase chain reaction [↑](#footnote-ref-12)
13. Tube feeding [↑](#footnote-ref-13)
14. Stratified block randomization [↑](#footnote-ref-14)
15. Numbered containers [↑](#footnote-ref-15)
16. Superoxide dismutase [↑](#footnote-ref-16)
17. Malondialdehyde [↑](#footnote-ref-17)
18. Total antioxidant capacity [↑](#footnote-ref-18)
19. Visual analogue scales  [↑](#footnote-ref-19)
20. A high-sensitivity c-reactive protein  [↑](#footnote-ref-20)
21. Analysis of covariance [↑](#footnote-ref-21)
22. Intention-to-treat [↑](#footnote-ref-22)
23. Last observation carried forward [↑](#footnote-ref-23)
